# Comprehensive bioinformatic analysis of the expression and prognostic significance of TSC22D domain family genes in adult acute myeloid leukemia

**DOI:** 10.1186/s12920-023-01550-7

**Published:** 2023-05-27

**Authors:** XiaoQiang Xu, Rui Sun, YuanZhang Li, JiaXi Wang, Meng Zhang, Xia Xiong, DanNi Xie, Xin Jin, MingFeng Zhao

**Affiliations:** 1 The First Central Clinical School, Tianjin Medical University, Tianjin, 300192 China; 2 Department of Hematology, Shanxi Fenyang Hospital, Fenyang, 032200 China; 3 School of Medicine, Nankai University, Tianjin, 300071 China; 4 Department of Hematology, Tianjin First Central Hospital, Tianjin, 300192 China

**Keywords:** Acute myeloid leukemia, Prognostic biomarker, Drug response, Tumor infiltration

## Abstract

**Background:**

TSC22D domain family genes, including TSC22D1-4, play a principal role in cancer progression. However, their expression profiles and prognostic significance in adult acute myeloid leukemia (AML) remain unknown.

**Methods:**

The online databases, including HPA, CCLE, EMBL-EBI, GEPIA2, BloodSpot, GENT2, UCSCXenaShiny, GSCALite, cBioportal, and GenomicScape, utilized the data of TCGA and GEO to investigate gene expression, mutation, copy number variation (CNV), and prognostic significance of the TSC22D domain family in adult AML. Computational analysis of resistance (CARE) was used to explore the effect of TSC22D3 expression on drug response. Functional enrichment analysis of TSC22D3 was performed in the TRRUST Version 2 database. The STRING, Pathway Commons, and AnimalTFDB3.0 databases were used to investigate the protein–protein interaction (PPI) network of TSC22D3. Harmonizome was used to predict target genes and kinases regulated by TSC22D3. The StarBase v2.0 and CancermiRNome databases were used to predict miRNAs regulated by TSC22D3. UCSCXenaShiny was used to investigate the correlation between TSC22D3 expression and immune infiltration.

**Results:**

Compared with normal adult hematopoietic stem cells (HSCs), the expression of TSC22D3 and TSC22D4 in adult AML tissues was markedly up-regulated, whereas TSC22D1 expression was markedly down-regulated. The expression of TSC22D1 and TSC22D3 was significantly increased in adult AML tissues compared to normal adult tissues. High TSC22D3 expression was significantly associated with poor overall survival (OS) and event-free survival (EFS) in adult AML patients. Univariate and multivariate Cox analysis showed that overexpression of TSC22D3 was independently associated with adverse OS of adult AML patients. High TSC22D3 expression had a adverse impact on OS and EFS of adult AML patients in the chemotherapy group. TSC22D3 expression correlated with drug resistance to BCL2 inhibitors. Functional enrichment analysis indicated that TSC22D3 might promote AML progression. MIR143-3p sponging TSC22D3 might have anti-leukemia effect in adult AML.

**Conclusions:**

A significant increase in TSC22D3 expression was observed in adult AML tissues compared to normal adult HSCs and tissues. The prognosis of adult AML patients with high TSC22D3 expression was unfavorable, which could severe as a new prognostic biomarker and potential target for adult AML.

**Supplementary Information:**

The online version contains supplementary material available at 10.1186/s12920-023-01550-7.

## Introduction

**Fig. 1:**
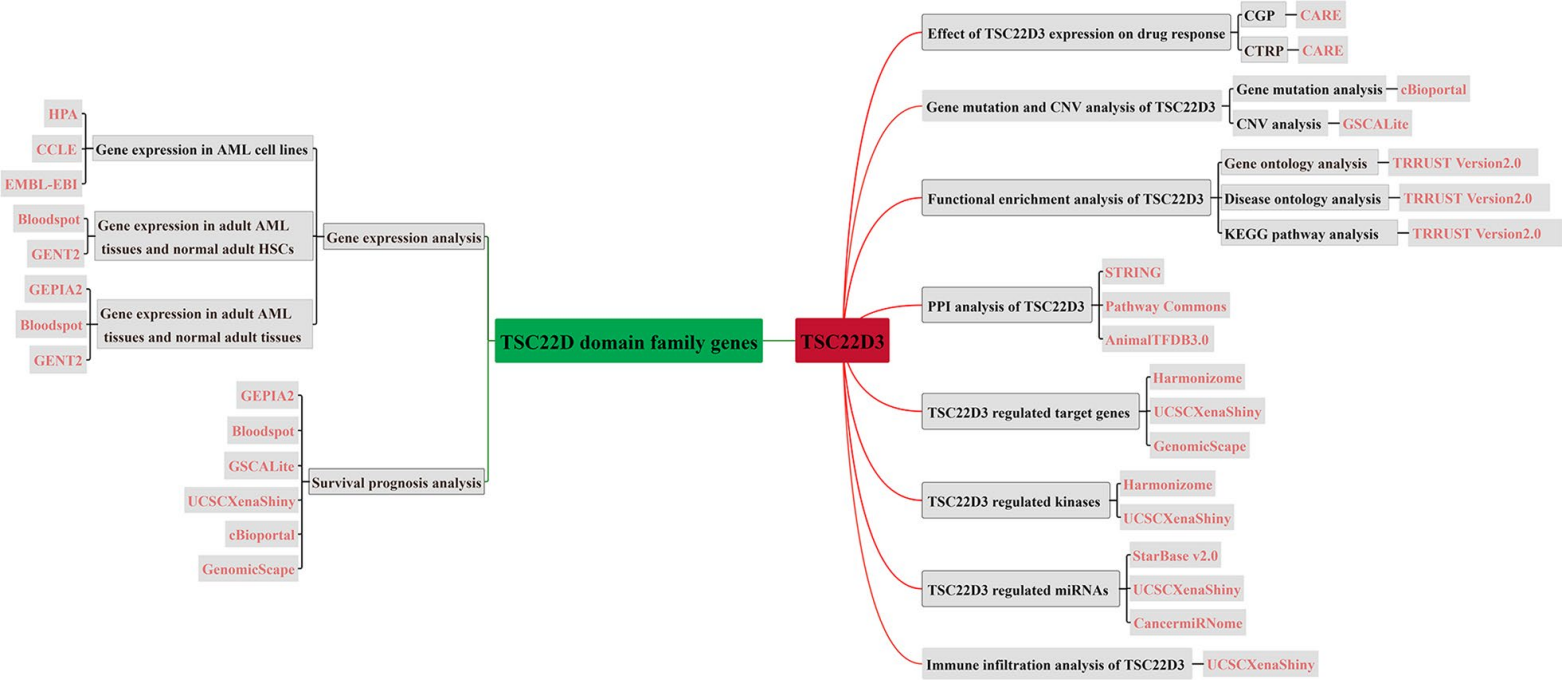
Flow chart of the present study

Acute myeloid leukemia (AML) is an aggressive hematopoietic malignancy with high biological and clinical heterogeneity [1]. Despite advances made in the diagnosis and treatment of AML, the increased risk of relapse and low 5-year survival rate after diagnosis remain significant challenges [2]. Authentication of new AML biomarkers can help to clarify the pathogenesis of the disease and guide the diagnosis, treatment, and prognosis evaluation of AML [3]. TSC22D domain family genes have been extensively reported to play an essential role in tumors [4–7]. Nonetheless, their expression profiles and prognosis in adult AML remain unclear. Herein, we conducted an integrated analysis of the expression and prognostic value of TSC22D domain family genes in adult AML by using data from the Cancer Genome Atlas (TCGA) and Gene Expression Omnibus (GEO) databases. The flow chart of our study was shown in Fig. 1.

## Materials and methods

### Data retrieval and processing

#### Gene expression analysis

##### Gene expression of the TSC22D domain family in AML cell lines

Human Protein Atlas (HPA, https://www.proteinatlas.org) [8] is a comprehensive database of proteomics, transcriptomics, and systems biology data. The expression of the TSC22D domain family genes in 88 leukemia cell lines (including 38 AML cell lines) was determined and visualized using “HPA”.

Cancer Cell Line Encyclopedia (CCLE, https://www.broadinstitute.org/ccle) [9] is a multiomics online database that provides a large-scale transcriptome sequencing data for the study of human cancer cell lines. The expression data of TSC22D domain family genes in 43 AML cell lines was downloaded from the “Expression 22Q4 Public” dataset of the CCLE database and visualized by the cluster heatmap tool.

EMBL's European Bioinformatics Institute (EMBL-EBI, https://www.ebi.ac.uk) [10] is an integrated bioinformatics research database. The expression of the TSC22D domain family genes in 16 AML cell lines was determined and visualized using “EMBL-EBI”.

##### Gene expression of the TSC22D domain family in adult AML tissues and CD34 positive hematopoietic stem cells (HSCs) from normal adult bone marrow tissues

BloodSpot (http://servers.binf.ku.dk/bloodspot/) [11] is an online open data analysis platform that provides gene expression and survival prognosis data from TCGA and GEO databases. Gene expression data of the TSC22D domain family in adult AML tissues and CD34 positive HSCs from normal adult bone marrow tissues was downloaded from the “Normal hematopoiesis with AMLs” dataset and the “Bloodpool: AML samples with normal cells” dataset of the BloodSpot database.

Gene Expression database of Normal and Tumor tissues 2 (GENT2, http://gent2.appex.kr) [12] integrates publicly available expression profile microarray data from the GEO database to compare and analyze gene expression in normal and cancer patient tissues. Gene expression data of the TSC22D domain family in 2802 adult AML tissues and 17 CD34 positive HSCs from normal adult bone marrow tissues was downloaded from the “ GPL570 platform (HG-U133_Plus_2)” of the GENT2 database (See excel sheet 1 in the Additional file 1).

##### Gene expression of the TSC22D domain family in adult AML tissues and normal adult tissues

Gene Expression Profling Interactive Analysis 2 (GEPIA2, http://gepia2.cancer-pku.cn/) [13] is an updated and enhanced online publicly accessible database based on TCGA and Genotype-Tissue Expression (GTEx) databases for tumor and normal samples for gene expression analysis. The expression of the TSC22D domain family genes in 173 TCGA-LAML tissues and 70 GTEx-Normal tissues was compared and visualized using “GEPIA2”.

Gene expression data of the TSC22D domain family in 542 adult AML tissues and 73 normal adult.bone marrow tissues was downloaded from the “ Leukemia MILE Study” dataset (GSE13159) of the BloodSpot database.

Gene expression data of the TSC22D domain family in 2802 adult AML tissues and 134 normal adult bone marrow tissues was downloaded from the “ GPL570 platform (HG-U133_Plus_2)” of the GENT2 database (See excel sheet 1 in the Additional file 1).

#### Survival analysis

We retrieved and analyzed the RNAseq gene expression data of the TSC22D domain family and the corresponding clinical prognostic data in the GEPIA2, Bloodspot, GSCALite ( http://bioinfo.life.hust.edu.cn/web/GSCALite/) [14], UCSCXenaShiny (https://shiny.hiplot-academic.com/ucsc-xena-shiny) [15], cBioportal (https://www.cbioportal.org) [16], and GenomicScape (http://genomicscape.com/) [17] databases. Furthermore, RNASeq (RNA-seq V2 RSEM) gene expression data of TSC22D3 and the corresponding clinical prognostic data was downloaded from the “ TCGA-LAML, NEJM 2013” [18] dataset of the cBioPortal database (See excel sheet 2 in the Additional file 2). Then adult patients with AML were stratified into a low expression group and a high expression group based on TSC22D3 mRNA median expression. We explored the relationship between TSC22D3 expression and clinical parameters and performed the analyses of OS, EFS, and univariate and multivariate Cox OS.

#### Effect of TSC22D3 expression on drug response

Computational analysis of resistance [19] (CARE, http://care.dfci.harvard.edu/) is used to identify genomes and biomarkers of response to targeted therapies. A positive CARE score indicated that gene expression was associated with drug sensitivity, whereas a negative CARE score indicated drug resistance.

Data of the correlation between TSC22D3 expression and drug response was downloaded from the “Cancer Genome Project (CGP)” dataset and the “Cancer Therapeutics Response Portal (CTRP)” dataset of the CARE database and visualized by the arc link tool.

#### Gene mutation and copy number variation (CNV) analysis of TSC22D3

Gene mutation data and the corresponding survival data of of TSC22D3 in adult AML from the “ TCGA-LAML, PanCancer Atlas” dataset was analyzed and visualize using “cBioPortal”. And CNV data and the corresponding survival data of TSC22D3 in adult AML was analyzed and visualized using “GSCALite”.

#### Functional enrichment analysis of TSC22D3

Transcriptional Regulatory Relationships Unraveled by Sentence-based Text mining Version 2 (TRRUST Version 2, http://www.grnpedia.org/trrust/) [20] is an online, open database of human and mouse transcriptional regulatory networks. Gene ontology (GO) biological process, disease ontology (DO), and Kyoto Encyclopedia of Genes and Genomes (KEGG) pathway data associated with human TSC22D3 transcription factor (TF) was downloaded from “TRRUST Version 2” and then visualized by the bar with a color gredient tool.

#### Protein–protein interaction (PPI) analysis of TSC22D3

STRING (https://string-db.org) [21] is an online open database aimed at providing customized protein–protein networks.

Pathway Commons (http://www.pathwaycommons.org) [22] is an integrated platform of multiple database for predicting protein–protein interactions.

AnimalTFDB3.0 (http://bioinfo.life.hust.edu.cn/AnimalTFDB/) [23] is an online database aimed at providing the most comprehensive and accurate information for animal (including human) TFs and cofactors.

The relationship between TSC22D3 and other proteins was predicted and visualized using “String,” “Pathway Commons,” and “AnimalTFDB3.0.” The expression of potential protein in 173 TCGA-LAML tissues and 70 GTEx-Normal tissues was analyzed using “UCSCXenaShiny”. The correlation between TSC22D3 protein and potential protein was analyzed and visualized using “UCSCXenaShiny”. The effect of potential protein on OS of adult AML patients was analyzed and visualized using “GenomicScape”.

#### Analysis of TSC22D3 regulated target genes and kinases

Harmonizome (http://amp.pharm.mssm.edu/Harmonizome) [24] integrates many publicly available online databases to predict the functions of genes or proteins.

Data of TSC22D3 regulated target genes was downloaded from the “CHEA Transcription Factor Targets” dataset, the “ENCODE Transcription Factor Targets” dataset, and the “JASPAR Predicted Transcription Factor Targets” dataset of the Harmonizome database and visualized using the jvenn tool.

The expression of potential target gene in 173 TCGA-LAML tissues and 70 GTEx-Normal tissues was analyzed and visualized using “UCSCXenaShiny”. The correlation between TSC22D3 and potential target gene was analyzed and visualized using “UCSCXenaShiny”. The effect of potential target gene on OS of adult AML patients was analyzed and visualized using “GenomicScape”.

Data of the top 20 predicted kinases with a high Z score regulated by TSC22D3 was downloaded from the Harmonizome database and visualized using the circular heatmap tool. The expression of predicted kinases in 173 TCGA-LAML tissues and 70 GTEx-Normal tissues was analyzed and visualized using “UCSCXenaShiny”. The correlation between TSC22D3 and predicted kinases was analyzed and visualized using “UCSCXenaShiny”. The effect of predicted kinases on OS of adult AML patients was analyzed and visualized using “UCSCXenaShiny”.

#### Analysis of TSC22D3 regulated miRNAs

StarBase v2.0 ( https://starbase.sysu.edu.cn/) [25] integrates multiple online microRNA (miRNA) databases to explore miRNA interactions.

CancerMIRNome ( http://bioinfo.jialab-ucr.org/CancerMIRNome) [26] is an online database for interactive analysis and visualization of the miRNome spectrum in human cancer.

Data of the predicted miRNAs regulated by TSC22D3 was downloaded from the “PITA” dataset, the “microT” dataset, the “miRmap”dataset, the “miRanda”dataset, the “PicTar”dataset, and the “TargetScan” dataset of the StarBase v2.0 database and visualized using the jvenn tool. The correlation between the TSC22D3 expression and potential miRNA was analyzed and visualized using “StarBase v2.0” and “UCSCXenaShiny”. The effect of potential miRNA on OS of adult AML patients was analyzed and visualized using “UCSCXenaShiny” and “CancerMIRNome”. DO and KEGG pathway analysis of potential miRNA was performed and visualized using “CancerMIRNome”.

#### Immune infiltration analysis of TSC22D3

Data of the correlation between the TSC22D3 expression and immune cell infiltration in adult AML by using the “CIBERSORT” algorithm, the “QUANTISEQ” algorithm, the “MCPCOUNTER” algorithm, the “EPIC” algorithm, and the “XCELL” algorithm was downloaded from the UCSCXenaShiny database and visualized using the correlation analysis tool.

### Data analysis and visualization

Wilcoxon rank-sum test was used for comparative analysis of gene expression. Kaplan Meier survival analysis (including OS and EFS) was performed using the log-rank test. Univariate and multivariate Cox's survival analysis was performed using SPSS software version 21.0. Graph Pad Prism version 8 was used for chi-square test analysis. *P* value < 0.05 indicated a significance level.

Data visualization was performed using the cluster heatmap tool, the circular heatmap tool, box tool, KM survival curve tool, jvenn tool, bar with color gredient tool, arc link tool, and correlation analysis tool from the website (http://www.bioinformatics.com.cn).

## Results

### Analysis of the expression of the TSC22D domain family genes in AML cell lines, normal adult HSCs, adult AML tissues and normal adult tissues

**Fig. 2:**
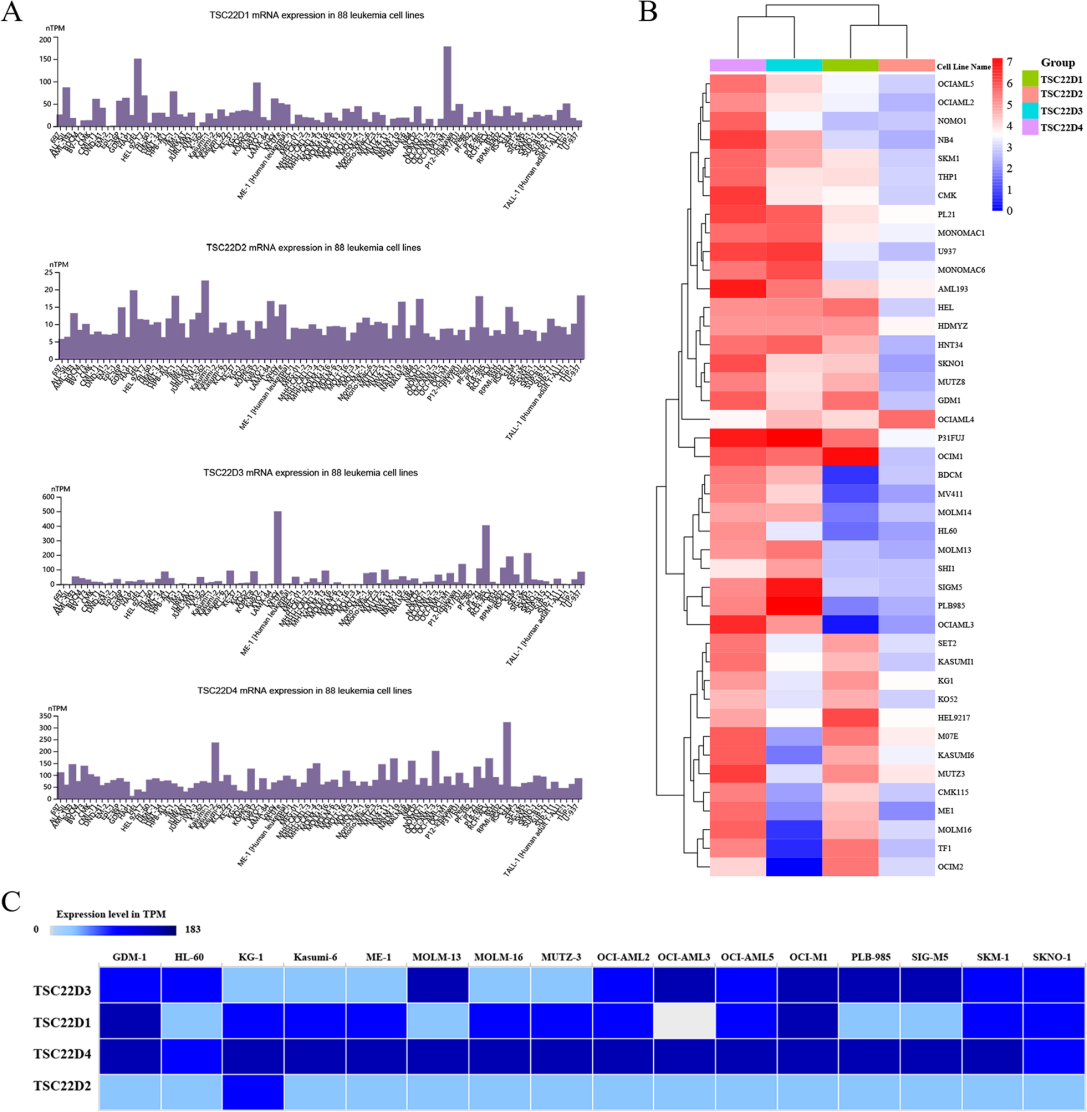
Gene expression of the TSC22D domain family in AML cell lines. **A** Bar graphs of the expression of the TSC22D domain family genes in 88 leukemia cell lines (including 38 AML cell lines) in the HPA database. The height of the bar charts represented the level of gene expression. **B** Heatmap of the expression of the TSC22D domain family genes in 43 AML cell lines in the CCLE database. The color of the Heatmap represented the level of gene expression. **C** Bar chart of the expression of the TSC22D domain family genes in 16 AML cell lines in the EMBL-EBI database. The shade of color in the bar graphs indicated the level of gene expression

Three different databases, including “HPA”, “CCLE”, and EMBL-EBI”, demonstrated that TSC22D domain family genes were abnormally expressed in AML cell lines at different levels (Fig. 2A–C).

**Fig. 3:**
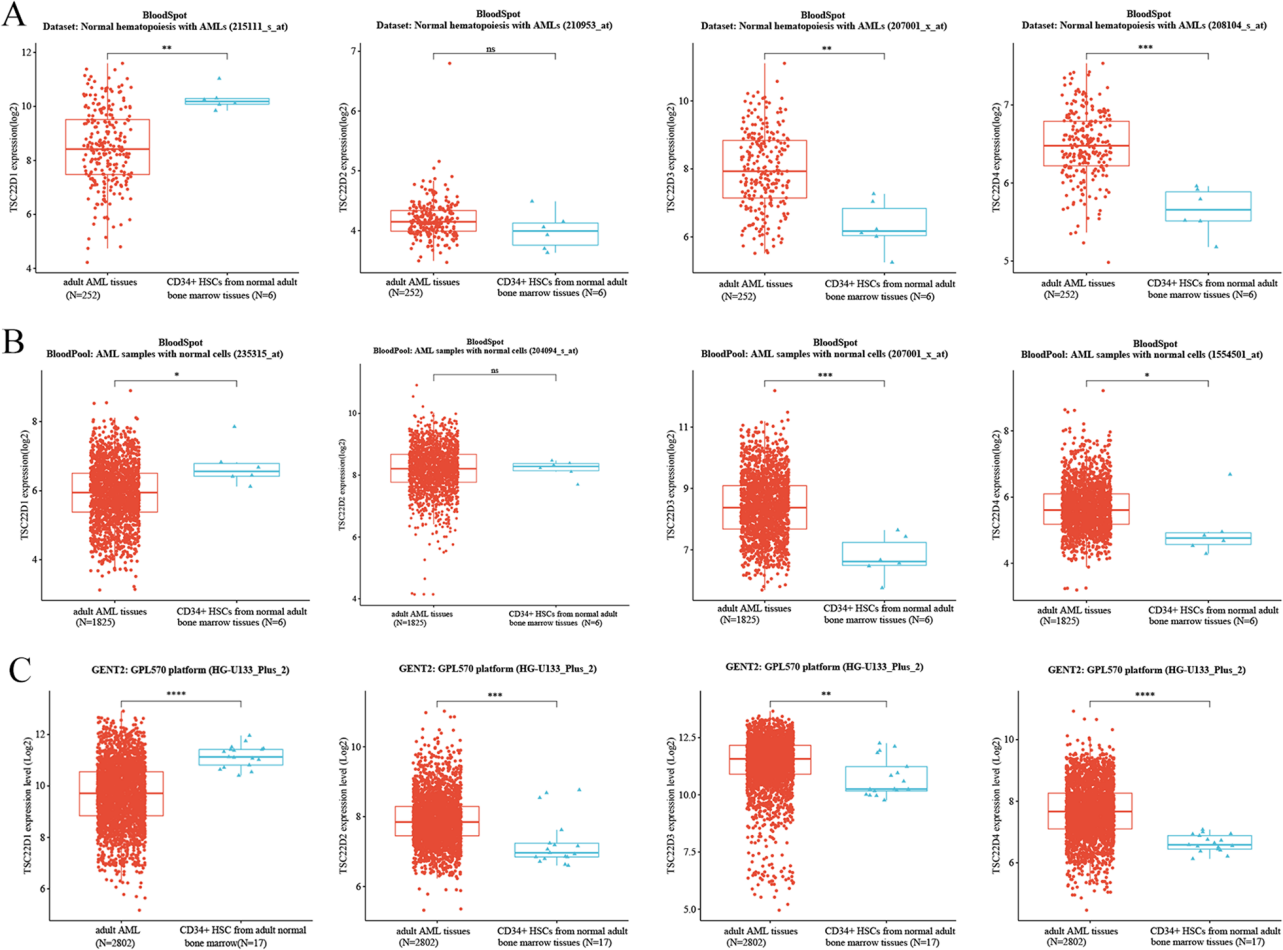
Gene expression of the TSC22D domain family in AML tissues and CD34 positive HSCs from normal adult bone marrow tissues was measured and compared using the Wilcoxon rank-sum test (*P < 0.05,**P < 0.01,***P < 0.001,****P < 0.0001, ns means no statistical significance). **A** The expression of TSC22D family genes in 252 AML tissues and 6 CD34 positive HSCs from normal adult bone marrow tissues was measured using the “ Normal hematopoiesis with AMLs” dataset of the Bloodspot database. **B** The expression of TSC22D family genes in 1825 AML tissues and 6 CD34 positive HSCs from normal adult bone marrow tissues was measured using the “ BloodPool: AML samples with normal cells” dataset of the Bloodspot database. **C** The expression of TSC22D family genes in 2802 AML tissues and 17 CD34 positive HSCs from normal adult bone marrow tissues was measured using the GPL570 platform (HG-U133_Plus_2) of the GENT2 database

Then we explored the expression of the TSC22D domain family genes in adult AML tissues and normal adult HSCs using TCGA and GEO data from the BloodSpot and GENT2 databases. These results showed that the expression of TSC22D3 and TSC22D4 was significantly upregulated in adult AML tissues relative to normal adult HSCs, whereas the expression trend of TSC22D1 was the opposite (*P* < 0.05) (Fig. 3A–C).

**Fig. 4:**
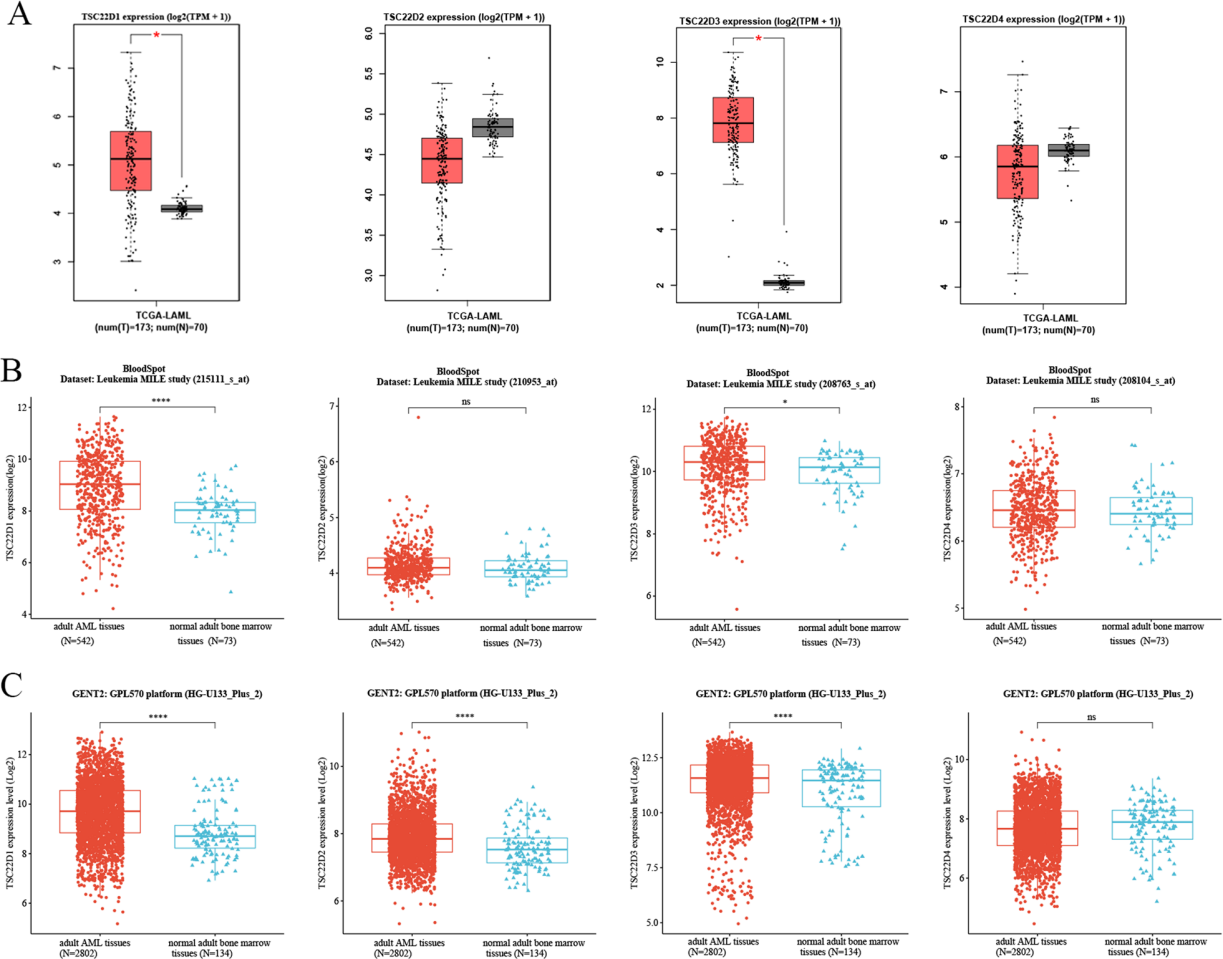
The expression of the TSC22D domain family genes in adult AML tissues and normal adult tissues was measured and compared using the Wilcoxon rank-sum test (*P < 0.05,**P < 0.01,***P < 0.001,****P < 0.0001, ns means no statistical significance). **A** The expression of the TSC22D domain family genes in 173 TCGA-LAML tissues and 70 GTEx-normal tissues using the GEPIA2 database. **B** The expression of the TSC22D domain family genes in 542 adult AML tissues and 73 normal adult bone marrow tissues using the Leukemia MILE study dataset of the Bloodspot database. **C** The expression of the TSC22D domain family genes in 2802 adult AML tissues and 134 normal adult bone marrow tissues using the GENT2 database

We subsequently investigated gene expression of the TSC22D domain family in adult AML tissues and normal adult tissues utilizing TCGA data from the GEPIA2 database, and GEO data from the BloodSpot and GENT2 databases. The results revealed that the expression of TSC22D1 and TSC22D3 was markedly increased in adult AML tissues compared with normal adult tissues (*P* < 0.05) (Fig. 4A–C).

### Survival analysis according to the expression of the TSC22D domain family genes in adult AML

Survival analysis was performed using TCGA data from the GEPIA2, Bloodspot, GSCALite, UCSCXenaShiny, and cBioportal databases and GEO data from the GenomicScape database. Amusingly, only TSC22D3 expression was of survival prognostic significance in adult AML. However, other members of the TSC22D family genes had little effect on OS of adult AML patients (See Table 1).

**Table 1:** The effect of the expression of the TSC22D domain family genes on OS of adult AML patients

Database	Adult AML samples (N)	Group	TSC22D1	TSC22D2	TSC22D3	TSC22D4
GEPIA2	106	Cutoff high value	0.50	0.50	0.50	0.50
		Logrank *P*-value	0.11	0.30	0.047	0.87
		Prognostic outcome	NS	NS	Adverse	NS
Bloodspot	172	Cutoff high value	0.50 * (probe ID: 215111_s_at)	0.49 * (probe ID: 210953_at)	0.51 * (probe ID: 208763_s_at)	0.49 * (probe ID: 208104_s_at)
		Logrank *P*-value	0.353	0.085	4.27E-03	0.68
		Prognostic outcome	NS	NS	Adverse	NS
GSCALite	163	Cutoff high value	0.50	0.50	0.50	0.50
		Logrank *P*-value	0.66	0.61	1.8E-03	0.77
		Prognostic outcome	NS	NS	Adverse	NS
UCSCXenaShiny	161	Cutoff high value	0.50	0.50	0.50	0.50
		Logrank *P*-value	0.31	0.14	1.7E-02	0.64
		Prognostic outcome	NS	NS	Adverse	NS
UCSCXenaShiny	167	Cutoff high value	0.49	0.51	0.50	0.50
		Logrank *P*-value	0.26	0.11	2.3E-03	0.48
		Prognostic outcome	NS	NS	Adverse	NS
cBioportal: Firehose Legacy	169	Cutoff high value	0.50	0.50	0.50	0.50
		Logrank *P*-value	0.155	0.139	3.0E-03	0.534
		Prognostic outcome	NS	NS	Adverse	NS
cBioportal: TCGA-NEJM2013	173	Cutoff high value	0.50	0.50	0.50	0.50
		Logrank *P*-value	0.855	0.326	0.041	0.323
		Prognostic outcome	NS	NS	Adverse	NS
cBioportal: TCGA PanCancer Atlas	161	Cutoff high value	0.50	0.50	0.50	0.50
		Logrank *P*-value	0.563	0.478	8.0E-03	0.759
		Prognostic outcome	NS	NS	Adverse	NS
GenomicScape	78	Cutoff high value	0.10 ^&^(probe ID: 243133_at)	0.91 ^&^(probe ID: 210954_s_at)	0.74 ^&^(probe ID: 235364_at)	0.91 ^&^(probe ID: 208104_s_at)
		Logrank *P*-value	0.11	0.072	0.025	0.20
		Prognostic outcome	NS	NS	Adverse	NS
GenomicScape:	162	Cutoff high value	0.83 ^&^(probe ID: 243133_at)	0.86 ^&^(probe ID: 210954_s_at)	0.20 ^&^(probe ID: 235364_at)	0.47 ^&^(probe ID: 208104_s_at)
		Logrank *P*-value	0.089	0.18	0.035	0.044
		Prognostic outcome	NS	NS	Adverse	Favorable

*NS* no significance

^*^Analysis of the effect of the TSC22D domain family genes on OS of adult AML patients was performed in the corresponding probe set of the Bloodspot database

^&^Analysis of the effect of the TSC22D domain family genes on OS of adult AML patients was performed in the corresponding probe set of the GenomicScape database

Morever, we found that high TSC22D3 expression was significantly correlated with white blood cell (WBC) counts (> 20 × 10^9/L), bone marrow (BM) blasts (> 70%), FAB M1 subtype, FAB M5 subtype, and positive NPM1 mutation (*P* < 0.05). Low TSC22D3 expression was significantly correlated with the FAB M2 subtype and the FAB M3 subtype (*P* < 0.05) (See Table 2).

**Table 2:** The relationship between TSC22D3 expression and clinical parameters in 173 adult AML data from the “TCGA-AML NEJM 2013” dataset of the cBioportal database

Characteristics	Low expression of TSC22D3	High expression of TSC22D3	*P*-value	Statistical approach
n	86	87		
Sex, n (%)			0.9354	Chi-square test
Male	46 (26.59%)	46 (26.59%)		
Female	40 (23.12%)	41(23.70%)		
Race, n (%)			0.6837	Chi-square with Yates' correction test
White	66 (48.53%)	62 (45.59%)		
Black	3 (2.20%)	5 (3.68%)		
Age, n (%)			0.1430	Chi-square test
≤ 60	53 (30.64%)	44(25.43%)		
> 60	33 (19.08%)	43 (24.85%)		
WBC count(× 10^9/L), n (%)			< 0.0001	Chi-square test
≤ 20	59 (34.10%)	32 (18.50%)		
> 20	27 (15.61%)	55 (31.79%)		
PB blasts(%), n (%)			0.1458	Chi-square test
< 20	40 (23.12%)	31 (17.92%)		
≥ 20	46 (26.59%)	56(32.37%)		
BM blasts(%), n(%)			0.0272	Chi-square test
≤ 70	46 (26.59%)	32 (18.50%)		
> 70	40 (23.12%)	55 (31.79%)		
FAB classifications, n (%)			0.0356	Chi-square test
M0	9 (5.26%)	7 (4.10%)		
M1	16 (9.36%)	28 (16.37%)		
M2	25 (14.62%)	13 (7.60%)		
M3	11 (6.43%)	5 (2.92%)		
M4	15 (8.77%)	19 (11.11%)		
M5	6 (3.51%)	12 (7.02%)		
M6	1 (0.59%)	1 (0.59%)		
M7	3 (1.75%)	0 (0%)		
Cytogenetics, n (%)			0.3945	Chi-square test
Normal	37(21.64%)	43 (25.15%)		
Complex	7 (4.09%)	15(8.77%)		
t(15;17)	10 (5.85%)	5 (2.92%)		
t(8;21)	5 (2.92%)	2 (1.17%)		
t(9;22)	2 (1.17%)	1 (0.59%)		
inv(16)	4 (2.34%)	6 (3.51%)		
11q23	2 (1.17%)	2 (1.17%)		
+ 8	5 (2.92%)	3 (1.75%)		
− 7	1 (0.59%)	3 (1.75%)		
+ 21	2 (1.17%)	1 (0.59%)		
Other	10 (5.85%)	5 (2.92%)		
FLT3 mutation, n (%)			0.2570	Chi-square test
Negative	65 (37.57%)	59 (34.10%)		
Positive	21 (12.14%)	28 (16.19%)		
NPM1 mutation, n (%)			0.0465	Chi-square test
Negative	68 (39.31%)	57 (32.95%)		
Positive	18(10.40%)	30 (17.34%)		
DNMT3A mutation, n (%)			0.5053	Chi-square test
Negative	67 (38.73%)	64(36.99%)		
Positive	19 (10.98%)	23 (13.30%)		
IDH1 mutation, n (%)			0.1211	Chi-square test
Negative	81 (46.82%)	76 (43.93%)		
Positive	5 (2.89%)	11 (6.36%)		
IDH2 mutation, n (%)			0.0779	Chi-square test
Negative	81 (46.82%)	75 (43.35%)		
Positive	5 (2.89%)	12 (6.94%)		
TET2 mutation, n (%)			0.7691	Chi-square test
Negative	78 (45.09%)	80 (46.24%)		
Positive	8(4.62%)	7 (4.05%)		
TP53 mutation, n (%)			0.5926	Chi-square test
Negative	80 (46.24%)	79 (45.67%)		
Positive	6 (3.47%)	8 (4.62%)		
CEBPA mutation, n (%)			0.7565	Chi-square test
Negative	79 (45.66%)	81 (46.82%)		
Positive	7(4.05%)	6 (3.47%)		
Treatment type, n (%)			0.2532	Chi-square test
Chemotherapy	46 (26.59%)	54 (31.21%)		
Transplant	40 (23.12%)	33 (19.08%)		

**Fig. 5:**
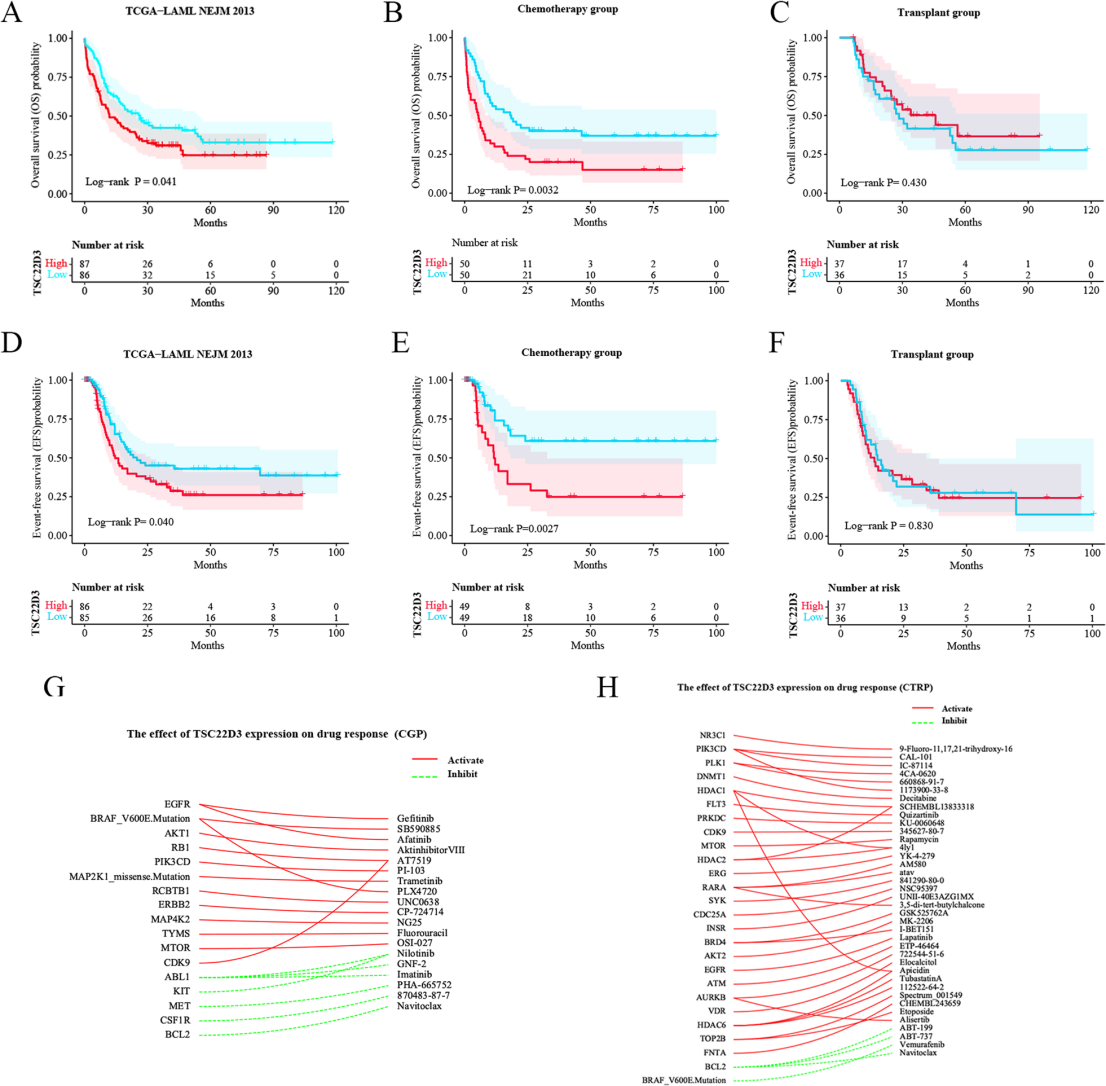
The effect of TSC22D3 expression on AML. **A** The effect of TSC22D3 expression on OS of 173 adult AML patients. **B** The effect of TSC22D3 expression on OS of 100 adult AML patients in the chemotherapy group. **C** The effect of TSC22D3 expression on OS of 73 adult AML patients in the transplantation group. **D** The effect of TSC22D3 expression on EFS of 171 adult AML patients. **E** The effect of TSC22D3 expression on EFS of 98 adult AML patients in the chemotherapy group. **F** The effect of TSC22D3 expression on EFS of 73 adult AML patients in the transplantation group. **G** The effect of TSC22D3 expression on drug response using the CGP dataset of the CARE database. **H** The effect of TSC22D3 expression on drug response using the CTRP dataset of the CARE database

We found that high TSC22D3 expression significantly affected OS and EFS of adult AML patients (*P* < 0.05) (Fig. 5A, D). Both univariate and multivariate COX regression analysis showed that the increased mortality in adult AML patients was significantly associated with over 60 years old, cytogenetics, DNMT3A positive mutation, TP53 positive mutation, treatment type (chemotherapy), and high TSC22D3 expression (*P* < 0.05) (See Table 3). High TSC22D3 expression had a detrimental effect on OS and EFS of adult AML patients in the chemotherapy group (*P* < 0.05) (Fig. 5B, E). However, high TSC22D3 expression had no effect on OS and EFS of adult AML patients in the transplantation group (*P* > 0.05) (Fig. 5C, F).

**Table 3:** Univariate and multivariate analysis of TSC22D3 expression and clinical parameters on OS of 173 adult AML patients from the “TCGA-AML NEJM 2013” dataset of the cBioportal database

Characteristics	Total(N)	Univariate analysis		Multivariate analysis	
		Hazard ratio (95% CI)	*P* value	Hazard ratio (95% CI)	*P* value
Age	173		< 0.001		
≤ 60	97	Reference		Reference	
> 60	76	3.131 (2.147–4.565)	< 0.001	2.047 (1.244–3.368)	0.005
Sex	173		0.770		
Male	92	Reference			
Female	81	1.056 (0.731–1.526)	0.770		
Race	136		0.628		
White	128	Reference			
Black	8	0.806 (0.328–1.983)	0.639		
WBC count (× 10^9/L)	173		0.291		
≤ 20	91	Reference			
> 20	82	1.219 (0.844–1.761)	0.290		
PB blast percentage	170		0.577		
< 20	68	Reference			
≥ 20	102	1.112 (0.764–1.620)	0.579		
Bone marrow blast percentage	173		0.451		
≤ 70%	78	Reference			
> 70%	95	1.153 (0.796–1.669)	0.452		
FAB	171		0.067		
M0	16	Reference			
M1	44	0.989 (0.495–1.974)	0.975		
M2	38	0.909 (0.447–1.848)	0.792		
M3	16	0.307 (0.106–0.887)	0.029		
M4	34	1.028 (0.506–2.090)	0.939		
M5	18	1.136 (0.501–2.577)	0.760		
M6	2	2.635 (0.578–12.017)	0.211		
M7	3	2.364 (0.654–8.543)	0.189		
Cytogenetics	171		0.005		0.019
Normal	80	Reference			
Complex	22	1.857 (1.088–3.171)	0.023	1.548 (0.716–3.346)	0.266
t(15;17)	15	0.360 (0.144–0.903)	0.029	0.400 (0.150–1.069)	0.068
t(8;21)	7	0.485 (0.152–1.553)	0.223	0.626 (0.184–2.130)	0.453
t(9;22)	3	2.266 (0.547–9.393)	0.259	5.015 (1.117–22.510)	0.035
inv(16)	10	0.308 (0.096–0.986)	0.047	0.373 (0.114–1.225)	0.104
t(11q23)	4	1.494 (0.466–4.791)	0.500	2.169 (0.659–7.141)	0.203
+ 8	8	1.231 (0.529–2.866)	0.630	1.303 (0.487–3.486)	0.598
− 7	4	1.672 (0.522–5.362)	0.387	2.253 (0.692–7.333)	0.177
+ 21	3	1.907(0.594–6.120)	0.278	3.493 (1.053–11.585)	0.041
Other	15	1.328(0.710–2.483)	0.375	1.920 (1.007–3.660)	0.047
FLT3 mutation	173		0.180		
Negative	124	Reference			
Positive	49	1.325 (0.885–1.984)	0.171		
NPM1 mutation	173		0.490		
Negative	125	Reference			
Positive	48	1.155 (0.770–1.732)	0.486		
DNMT3A mutation	173		0.038		
Negative	131	Reference		Reference	
Positive	42	1.571 (1.040–2.373)	0.032	1.675 (1.060–2.647)	0.027
IDH2 mutation	173		0.915		
Negative	156	Reference			
Positive	17	1.033 (0.567–1.884)	0.915		
IDH1 mutation	173		0.304		
Negative	157	Reference			
Positive	16	0.711 (0.360–1.406)	0.327		
TET2 mutation	173		0.991		
Negative	158	Reference			
Positive	15	0.996 (0.521–1.907)	0.991		
TP53 mutation	173		< 0.001		
Negative	159	Reference		Reference	
Positive	14	4.100 (2.291–7.339)	< 0.001	2.691 (1.145–6.323)	0.023
CEBPA mutation	173		0.829		
Negative	160	Reference			
Positive	13	0.928 (0.470–1.834)	0.831		
Treatment type	173		0.001		
Chemotherapy	100	Reference		Reference	
Transplant	73	0.519 (0.355–0.761)	0.001	0.466 (0.272–0.797)	0.005
TSC22D3 expression	173		0.042		
Low	86	Reference		Reference	
High	87	1.466 (1.012–2.122)	0.043	1.546 (1.031–2.320)	0.035

### Analysis of the effect of TSC22D3 expression on drug response

Analysis of the effect of TSC22D3 expression on drug response using the “CGP” dataset and the “CTRP” dataset demonstrated that TSC22D3 expression was significantly associated with drug resistance to BCL2 inhibitors (Fig. 5G, H).

### Gene mutation and CNV analysis of TSC22D3

**Fig. 6:**
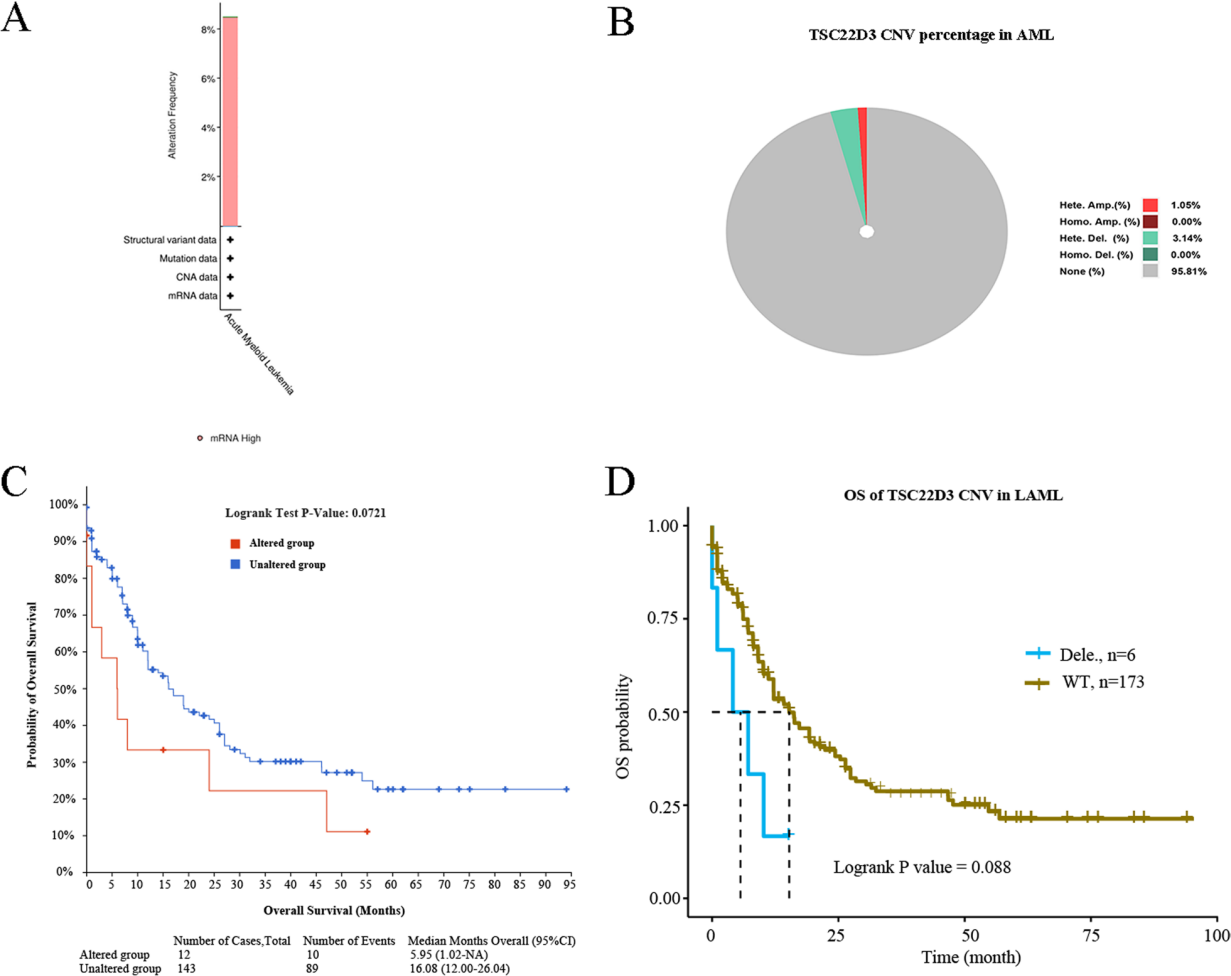
The profiles of gene mutation and CNV of TSC22D3 in adult AML and its effect on OS of adult AML patients. **A** Gene mutation rate of TSC22D3 in 165 adult AML samples using the TCGA PanCancer Atlas dataset of the cBioportal database. **B** The CNV of TSC22D3 in 179 adult AML samples using the GSCALite database. **C** The effect of gene mutation of TSC22D3 on OS of 165 adult AML patients using the cBioportal database. **D** The effect of CNV of TSC22D3 on OS of 179 adult AML patients using the GSCALite database

Gene mutation rate of TSC22D3 was 8%, and TSC22D3 gene mutation did not affect the OS of adult AML patients (Fig. 6A, C). An the incidence of CNV of TSC22D3 was low in adult AML and did not affect the OS of adult AML patients (Fig. 6B, D).

### Functional enrichment analysis of TSC22D3

**Fig. 7:**
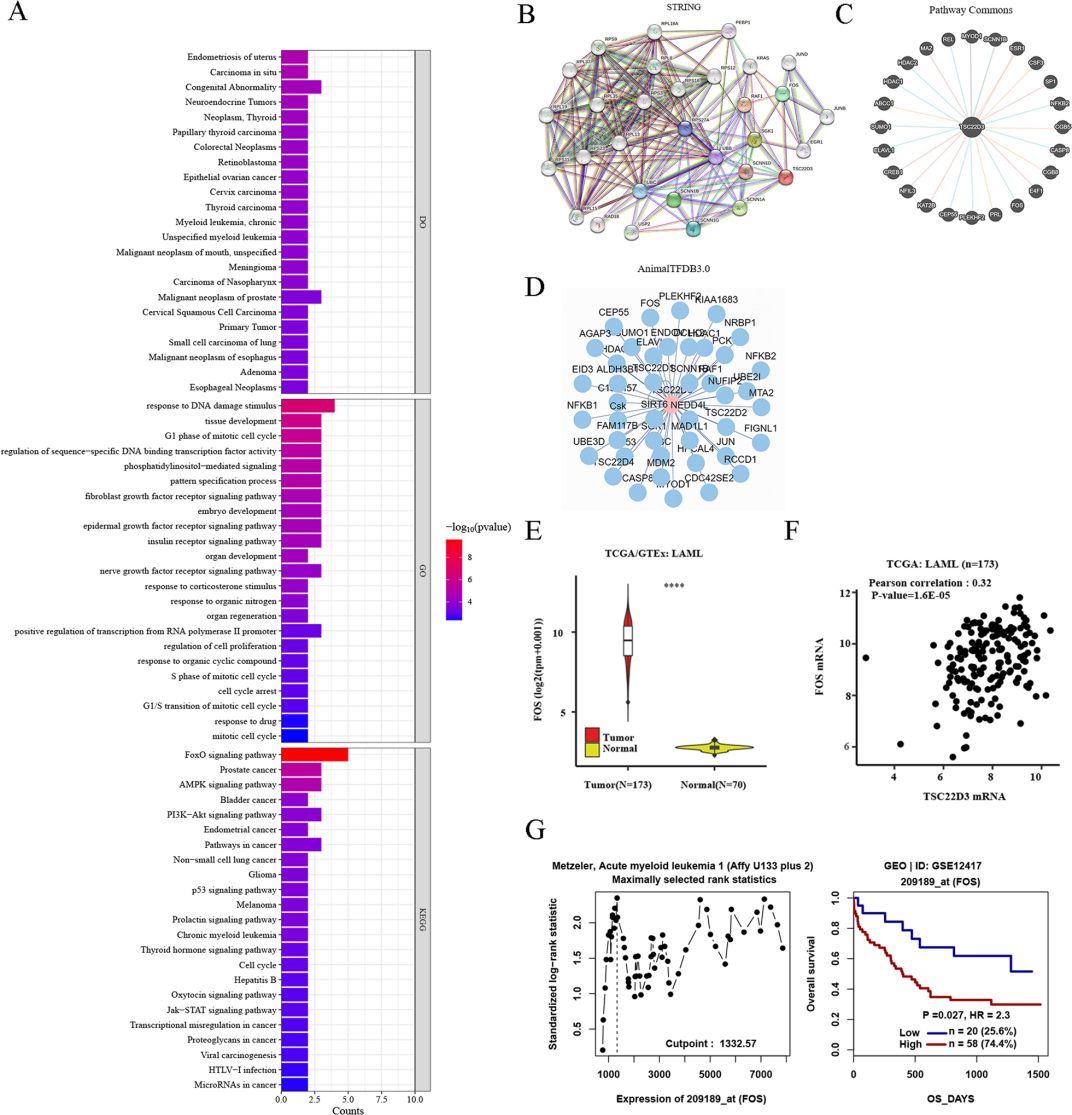
Functional enrichment analysis and PPI analysis of TSC22D3. **A** Gene ontology biological process, diseases ontology, and KEGG pathway of TSC22D3 using the TRRUST Version 2 database. **B** PPI analysis of TSC22D3 using the String database. **C** PPI analysis of TSC22D3 using the Pathway Commons database. **D** PPI analysis of TSC22D3 using the AnimalTFDB3.0 database. **E** The expression of FOS in 173 TCGA-LAML and 70 GTEx-Normal using the UCSCXenaShiny database. **F** The correlation between TSC22D3 and FOS using the UCSCXenaShiny database. **G** The effect of FOS expression on OS of adult AML patients using the GenomicScape database

The results showed that TSC22D3 has many biological functions, including response to DNA damage stimulus, G1 phase of mitotic cell cycle, regulation of cell proliferation, cell cycle arrest, and response to drug, etc. (Fig. 7A). DO analysis revealed that TSC22D3 was involved in tumors, including myeloid leukemia (Fig. 7A). Furthermore, KEGG pathway analysis indicated that TSC22D3 was involved in the regulation of multiple signaling pathways (Fig. 7A).

### PPI analysis of TSC22D3

The consistent analysis of the STRING, Pathway Commons, and AnimalTFDB3.0 databases indicated that TSC22D3 interacted with FOS and SCNN1B (Fig. 7B–D). And FOS has been extensively reported in tumor progression. Then the analysis of gene expression, correlation, and survival prognosis of FOS in adult AML was performed in TCGA and GEO datasets. The results revealed that FOS was significantly increased in adult AML (*P* < 0.05) (Fig. 7E). TSC22D3 was positively correlated with FOS (*P* < 0.05) (Fig. 7F). High FOS expression was associated with unfavorable OS of 78 adult AML patients (*P* < 0.05) (Fig. 7G).

### Analysis of TSC22D3 regulated target genes and kinases

**Fig. 8:**
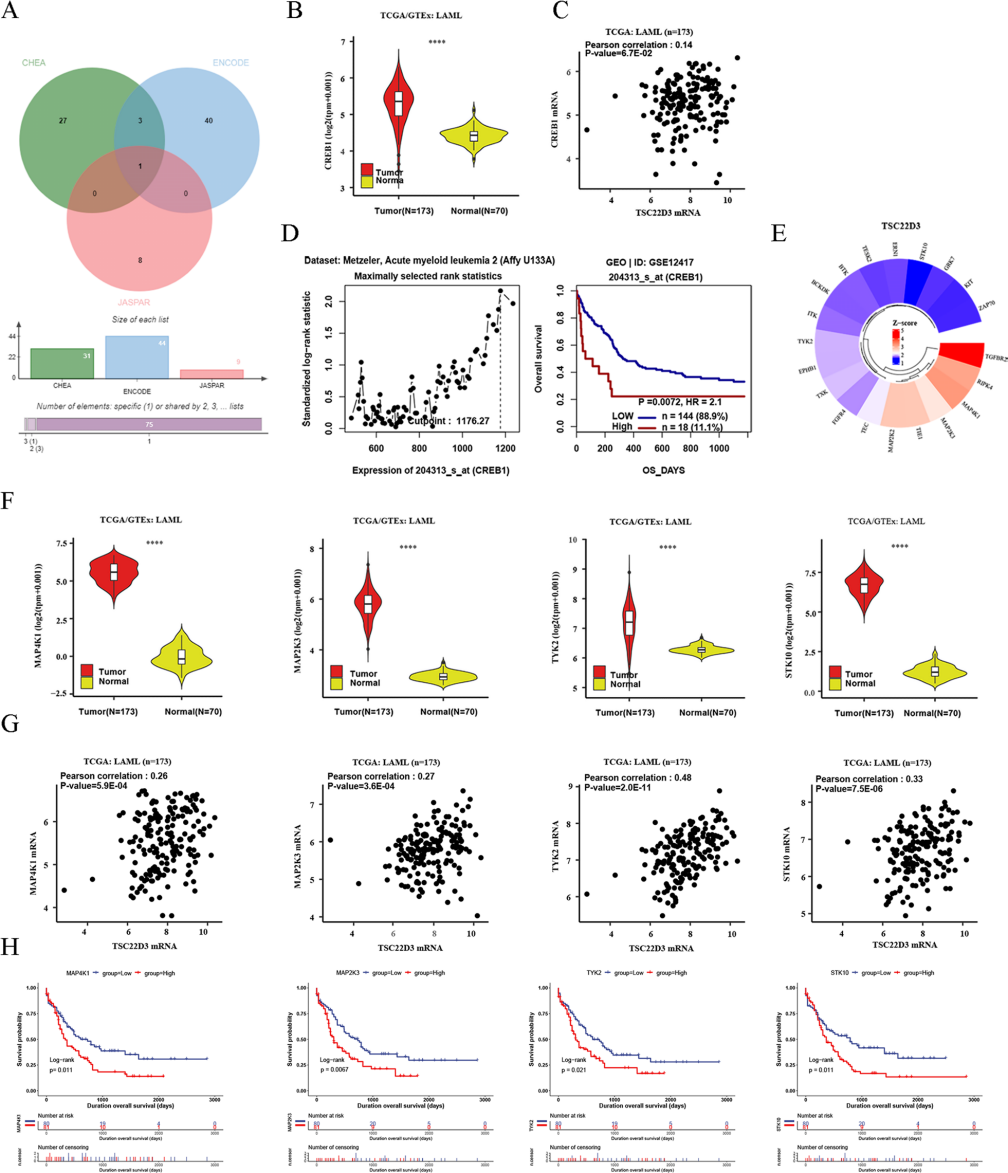
Predicted target genes and kinases regulated by TSC22D3. **A** The target genes regulated by TSC22D3 using the Harmonizome database. **B** The expression of CREB1 in 173 TCGA-LAML tissues and 70 GTEx-Normal tissues using the UCSCXenaShiny database. **C** The correlation between TSC22D3 and CREB1 using the UCSCXenaShiny database. **D** The effect of CREB1 expression on OS of 162 adult AML patients using the GenomicScape database. **E** Top 20 kinases regulated by TSC22D3 using the Harmonizome database. **F** The expression of predicted kinases in 173 TCGA-LAML tissues and 70 GTEx-Normal tissues using the UCSCXenaShiny database. **G** The correlation between TSC22D3 and predicted kinases using the UCSCXenaShiny database. **H** The effect of TSC22D3 regulated kinases on OS of 161 adult AML patients using the UCSCXenaShiny database

The results showed that TSC22D3 might regulate CREB1 (Fig. 8A). Gene expression analysis indicated that CREB1 was significantly elevated in adult AML (*P* < 0.05) (Fig. 8B). Unexpectedly, TSC22D3 had statistical no correlation with CREB1 (*P* > 0.05) (Fig. 8C). However, high CREB1 expression had an adverse impact on OS of 162 adult AML patients (*P* < 0.05) (Fig. 8D).

We investigated the top 20 kinases with high Z score regulated by TSC22D3 (Fig. 8E) and analyzed their expression, as well as the correlation of TSC22D3, and survival prognosis in adult TCGA-LAML (Additional file 3: See Table S1). The results revealed that the expression of MAP4K1, MAP2K3, TYK2, and STK10 was markedly up-regulated in adult AML and significantly positively correlated with TSC22D3 (*P* < 0.05) (Fig. 8F, G). And These kinases had an unfavorable effect on OS of 161 adult AML patients (*P* < 0.05) (Fig. 8H).

### Analysis of miRNAs regulated by TSC22D3

**Fig. 9:**
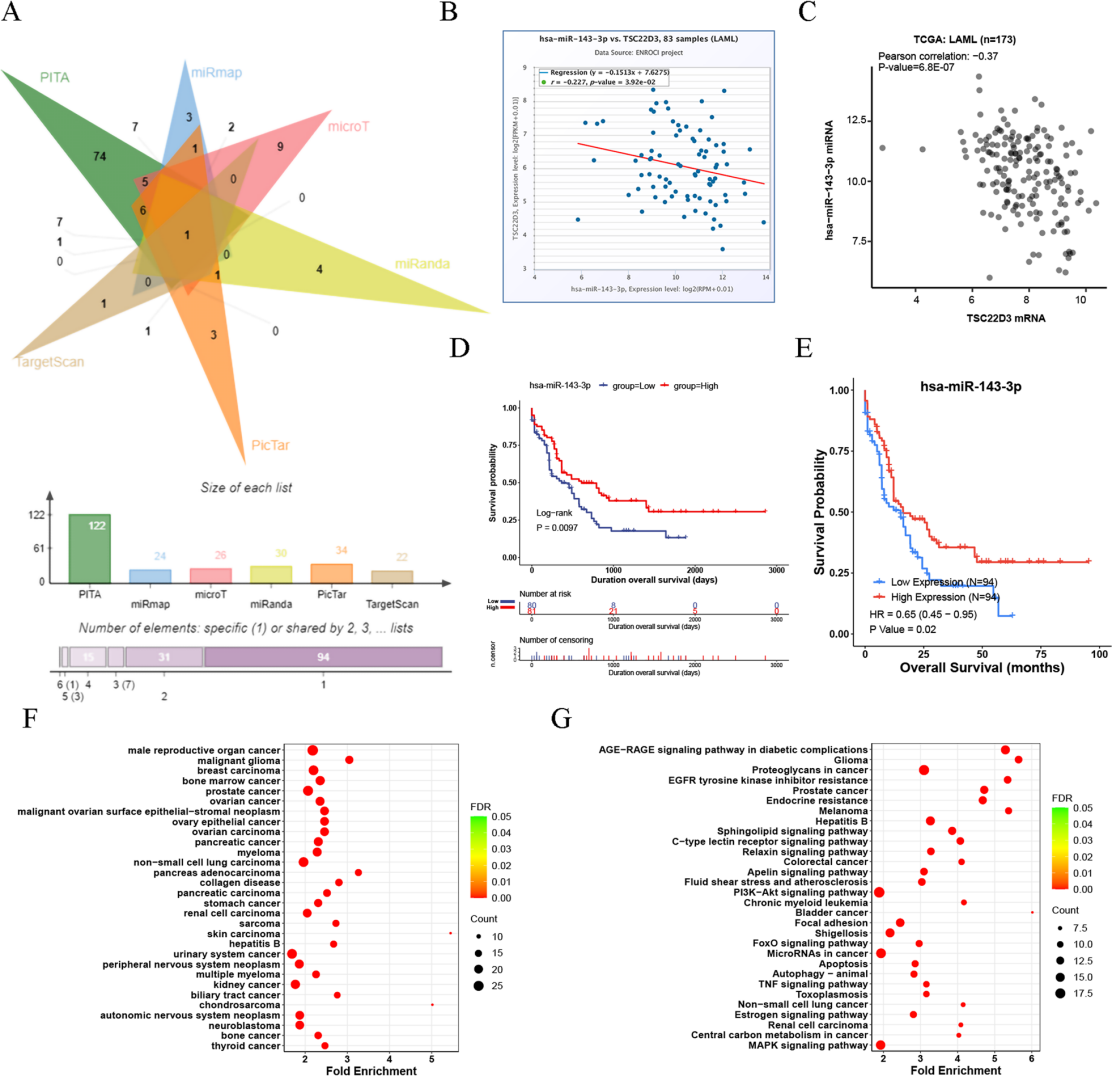
Analysis of miRNAs regulated by TSC22D3. **A** Predicted miRNAs regulated by TSC22D3 using the StarBase v2.0 database. **B** The correlation between TSC22D3 and MIR143-3p in 83 adult AML samples using the StarBase v2.0 database. **C** The correlation between TSC22D3 and MIR143-3p in 173 adult AML samples using the UCSCXenaShiny database. **D** The effect of MIR143-3p expression on OS of 161 adult AML patients using the UCSCXenaShiny database. **E** The effect of MIR143-3p expression on OS of 188 adult AML patients using the CancermiRNome database. **F** Diseases ontology of MIR143-3p using the CancermiRNome database. **G** KEGG pathways of MIR143-3p using the CancermiRNome database

Analysis of six different miRNA datasets revealed that TSC22D3 might be a possible target gene of MIR143-3p (Fig. 9A). TSC22D3 was negatively correlated with MIR143-3p (*P* < 0.05) (Fig. 9B, C). High expression of MIR143-3p was a favorable prognostic factor for OS of adult AML patients (*P* < 0.05) (Fig. 9D, E). DO and KEGG analysis indicated that MIR143-3p was involved in bone marrow cancer, including myeloid leukemia (Fig. 9F, G).

### Immune infiltration analysis of TSC22D3

**Fig. 10:**
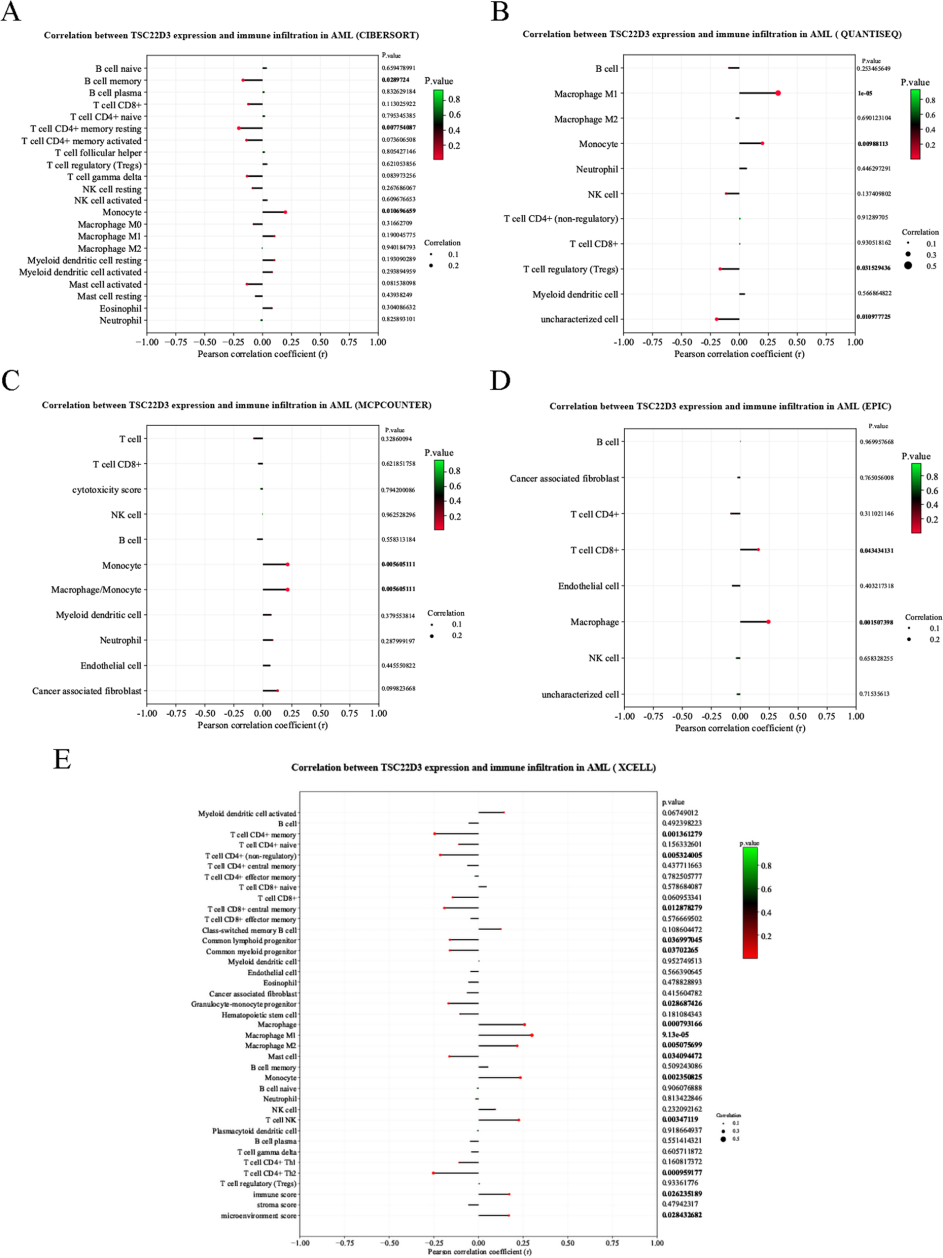
The correlation between TSC22D3 expression and immune cell infiltration in adult AML using the UCSCXenaShiny database. **A** CIBERSORT **B** QUANTISEQ **C** MCPCOUNTER **D** EPIC **E** XCELL

Analysis of the correlation of TSC22D3 expression and immune cell infiltration in adult AML by using five different algorithms showed that TSC22D3 expression was significantly associated with monocyte/macrophage (*P* < 0.05) (Fig. 10A–E).

## Discussion

TSC22D domain family genes, including TSC22D1-4, belong to the leucine zipper TF family and have been reported to be involved in regulating cell proliferation and differentiation [27]. TSC22D1, also called transforming growth factor-β-stimulated clone-22, was reported to play a tumor suppressor role in tumors [28]. TSC22D2 depends on the TSC22D2-PKM2-CyclinD1 regulatory axis to inhibit tumor cell growth in colorectal cancer [29]. TSC22D3, also known as glucocorticoid-induced leucine zipper (GILZ), can promote or suppress tumor growth, depending on the type of tumor and its microenvironment. TSC22D3 plays a dual role in tumors: it not only exerts a tumor-promoting effect by influencing the immune system and tumor microenvironment but also inhibits tumor growth by inducing apoptosis or suppressing the proliferation of cancer cells [30]. TSC22D4, also known as THG-1, was reported to promote esophageal squamous cell carcinoma cell tumorsphere growth [31].

In our study, TCGA and GEO data was used to investigate the expression of TSC22D domain family genes and their prognostic significance in adult AML. These results showed that the expression of TSC22D1 and TSC22D3 was markedly increased in adult AML tissues. Stunningly, it was TSC22D3, not other TSC22D family genes, that had prognostic significance for OS of adult AML patients. Therefore, we focused on the possible role of TSC22D3 in adult AML. Our study revealed that adult AML patients with high expression of TSC22D3 had adverse OS and EFS. And overexpression of TSC22D3 was an independently survival prognostic factor in adult AML patients. Subgroup survival analysis according to treatment type also showed that high TSC22D3 expression was significantly associated with unfavorable OS and EFS in the chemotherapy group. However, we found no effect of TSC22D3 expression on OS and EFS of adult AML patients in the transplantation group. This suggested that transplantation might overcome the disadvantages of TSC22D3 expression. Furthermore, we found that high TSC22D3 expression was significantly associated with high WBC counts, high BM blasts, FAB M1 subtype, FAB M5 subtype, and positive mutation of NPM1. This partly explained why high expression of TSC22D3 was associated with a poor survival prognosis in adult AML.

Hyperactivation of BCL2 is associated with the development, progression, prognosis, and resistance to chemotherapy in AML. BCL2 inhibitors including Venetoclax have been applied in the clinical treatment of AML [32]. However, with the widespread use of Venetoclax, drug resistance has gradually emerged in AML patients, especially in the relapsed/refractory AML patients. Preclinical and clinical studies have partially unraveled the mechanism of drug resistance to Venetoclax [33]. The clinical application of BCL2 inhibitors still faces many challenges, which may be relevant to the fact that the complex mechanism of drug resistance has not been fully unraveled. Fascinatingly, our study showed that TSC22D3 expression was significantly correlated with resistance to BCL2 inhibitors. This might be one of the reasons for drug resistance of BCL2 inhibitors, and it was worth further exploring the underlying mechanism of drug resistance mediated by TSC22D3.c-Fos has been reported to play crucial parts in the maintenance and proliferation of AML [34]. Interestingly, our study indicated that TSC22D3 might transcriptionally up-regulate the expression of FOS, which might play a certain role in AML progression. TSC22D3 promoted tumor cell proliferation by regulating AKT kinase [35]. And hyperactivity of the kinases was involved in cancer progression. Therefore, we analyzed the kinases regulated by TSC22D3. Our study indicated that TSC22D3 might transcriptionally activate the kinases of MAP4K1, MAP2K3, TYK2, and STK10. MAP4K1, as an oncogene, promoted AML progression by regulating the cell cycle through the MAPK pathway [36]. MAP2K3 promoted tumor progression by regulating tumor cell migration and invasion through the JNK signaling pathway [37]. Dysregulated activation of TYK2 in cancers may lead to hyperactive JAK/STATs signal, which may play an important role in the occurrence and development of cancers [38]. The prognosis of AML patients with expressing high levels of STK10 was poor, which could severe as a new prognostic biomarker for AML [39].

MIR143-3p has been reported to function as a tumor suppressor [40]. Our study indicated that MIR143-3p might exhibit anti-leukemic effect by downregulating the expression of TSC22D3. TSC22D3 has been reported to be involved in the supervision of the cell cycle, differentiation, and apoptosis of immune cells [41]. TSC22D3 may play an anti-inflammatory and immunosuppressive role in tumor development. Activation of the immunosuppressive TSC22D3 TF in dendritic cells can result in treatment failure [42]. Overexpression of TSC22D3 subverted therapy-induced anticancer immuno-surveillance [43]. As a TF, TSC22D3 may mediate the immunosuppressive and anti-inflammatory effects of T cells and macrophages by inhibiting nuclear factor-κB (NF-κB)-dependent transcription [44, 45]. Furthermore, TSC22D3 played a significant role in tumor progression by mediating the increase in cell quantity and activity of Treg cells through the TGF-β signaling pathway [46, 47]. TSC22D3 could play an indispensable role in the tumor microenvironment by influencing all immune system cells that infiltrated the tumor microenvironment [30]. In addition, TSC22D3 may serve as a pivotal regulator of T cell predysfunction [48]. Recent research shows that the proliferation, survival, and drug resistance of AML cells may be sustained and modulated by the bone marrow immunosuppressive microenvironment [49]. Our study showed a significantly positive correlation between monocyte/macrophage and TSC22D3 expression. How did TSC22D3 regulate monocyte/macrophage needed further study in AML immune microenvironment.

To sum up, TSC22D3 might be involved in AML progression through multiple mechanisms, including the regulation of target genes, kinases, signaling pathways, drug resistance, and immune cell infiltration. MIR143-3p sponging TSC22D3 might exhibit anti-leukemic effect in adult AML. Our study extended our understanding of TSC22D3 as a novel prognostic factor in adult AML and its potential role in AML.

## Supplementary Information

**Additional file 1**. The expression of the TSC22D domain family genes in normal adult HSCs, normal adult tissues, and adult AML tissues using the Gent2 database.

**Additional file 2**. The RNASeqexpression data of TSC22D3 and the corresponding clinical prognostic data using the “ TCGA-LAML, NEJM 2013” dataset of the cBioPortal database.

**Additional file 3: Table S1**. Analysis of the expression, correlation, and survival prognosis of predicted kinases regulated by TSC22D3 using the UCSCXenaShiny and Harmonizome database.

## Data Availability

The datasets provided for this study can be found and accessed in online databases. These online databases were accessible from the following addresses. HPA, https://www.proteinatlas.org; CCLE, https://www.broadinstitute.org/ccle; EMBL-EBI, https://www.ebi.ac.uk; BloodSpot, http://servers.binf.ku.dk/bloodspot/; GENT2, http://gent2.appex.kr; GEPIA2, http://gepia2.cancer-pku.cn/; GSCALite, http://bioinfo.life.hust.edu.cn/web/GSCALite/; UCSCXenaShiny, https://shiny.hiplot-academic.com/ucsc-xena-shiny; cBioportal, https://www.cbioportal.org; GenomicScape, http://genomicscape.com/; CARE, http://care.dfci.harvard.edu/; TRRUST Version 2, http://www.grnpedia.org/trrust/; STRING, https://string-db.org; Pathway Commons, http://www.pathwaycommons.org; AnimalTFDB3.0, http://bioinfo.life.hust.edu.cn/AnimalTFDB/; Harmonizome, http://amp.pharm.mssm.edu/Harmonizome; StarBase v2.0, https://starbase.sysu.edu.cn/; CancerMIRNome, http://bioinfo.jialab-ucr.org/CancerMIRNome.

## Abbreviations

AML or LAML: Acute myeloid leukemia
HPA: Human Protein Atlas
CCLE: Cancer Cell Line Encyclopedia
EMBL-EBI: EMBL’s European Bioinformatics Institute
GEPIA2: Gene Expression Profling Interactive Analysis 2
GENT2: Gene Expression database of Normal and Tumor tissues 2
CARE: Computational analysis of resistance
CGP: Cancer Genome Project
CTRP: Cancer Therapeutics Response Portal
TRRUST: Transcriptional Regulatory Relationships Unraveled by Sentence-based Text mining
CNV: Copy number variation
TCGA: The Cancer Genome Atlas
GEO: Gene Expression Omnibus
PPI: Protein–protein interaction
KEGG: Kyoto Encyclopedia of Genes and Genomes
GTEx: Genotype-Tissue Expression
TF: Transcription factor
OS: Overall survival
EFS: Event-free survival
GILZ: Glucocorticoid-induced leucine zipper
HSC: Hematopoietic stem cell
GO: Gene ontology
DO: Disease ontology
miRNA: MicroRNA
WBC: White blood cell
BM: Bone marrow
FAB: French–American–British

## References

[1] Döhner H, Weisdorf DJ, Bloomfield CD (2015). Acute myeloid leukemia. N Engl J Med.

[2] Doucette K, Karp J, Lai C (2021). Advances in therapeutic options for newly diagnosed, high-risk AML patients. Ther Adv Hematol.

[3] Prada-Arismendy J, Arroyave JC, Röthlisberger S (2017). Molecular biomarkers in acute myeloid leukemia. Blood Rev.

[4] Meijer D, Jansen MP, Look MP (2009). TSC22D1 and PSAP predict clinical outcome of tamoxifen treatment in patients with recurrent breast cancer. Breast Cancer Res Treat.

[5] Xiao L, Wei F, Liang F (2019). TSC22D2 identified as a candidate susceptibility gene of multi-cancer pedigree using genome-wide linkage analysis and whole-exome sequencing. Carcinogenesis.

[6] Qadir F, Aziz MA, Sari CP (2018). Transcriptome reprogramming by cancer exosomes: identification of novel molecular targets in matrix and immune modulation. Mol Cancer.

[7] Zhang X, Koga N, Suzuki H (2020). Promotion of cellular senescence by THG-1/TSC22D4 knockout through activation of JUNB. Biochem Biophys Res Commun.

[8] Thul PJ, Lindskog C (2018). The human protein atlas: a spatial map of the human proteome. Protein Sci.

[9] Ghandi M, Huang FW, Jané-Valbuena J (2019). Next-generation characterization of the Cancer Cell Line Encyclopedia. Nature.

[10] Madeira F, Park YM, Lee J (2019). The EMBL-EBI search and sequence analysis tools APIs in 2019. Nucleic Acids Res.

[11] Bagger FO, Kinalis S, Rapin N (2019). BloodSpot: a database of healthy and malignant haematopoiesis updated with purified and single cell mRNA sequencing profiles. Nucleic Acids Res.

[12] Park SJ, Yoon BH, Kim SK (2019). GENT2: an updated gene expression database for normal and tumor tissues. BMC Med Genom.

[13] Tang Z, Kang B, Li C (2019). GEPIA2: an enhanced web server for large-scale expression profiling and interactive analysis. Nucleic Acids Res.

[14] Liu CJ, Hu FF, Xia MX (2018). GSCALite: a web server for gene set cancer analysis. Bioinformatics.

[15] Wang S, Xiong Y, Zhao L (2022). UCSCXenaShiny: an R/CRAN package for interactive analysis of UCSC Xena data. Bioinformatics.

[16] Gao J, Aksoy BA, Dogrusoz U, et al. Integrative analysis of complex cancer genomics and clinical profiles using the cBioPortal. Sci Signal. 2013;6(269):pl1. 10.1126/scisignal.2004088. 10.1126/scisignal.2004088 PMC4160307 23550210

[17] Kassambara A, Rème T, Jourdan M, et al. GenomicScape: an easy-to-use web tool for gene expression data analysis. Application to investigate the molecular events in the differentiation of B cells into plasma cells. PLoS Comput Biol. 2015;11(1):e1004077. 10.1371/journal.pcbi.1004077. 10.1371/journal.pcbi.1004077 PMC4310610 25633866

[18] Cancer Genome Atlas Research Network; Ley TJ, Miller C, Ding L, et al. Genomic and epigenomic landscapes of adult de novo acute myeloid leukemia. N Engl J Med. 2013;368(22):2059–2074. 10.1056/NEJMoa1301689. 10.1056/NEJMoa1301689 PMC3767041 23634996

[19] Jiang P, Lee W, Li X (2018). Genome-scale signatures of gene interaction from compound screens predict clinical efficacy of targeted cancer therapies. Cell Syst.

[20] Han H, Cho JW, Lee S, et al. TRRUST v2: an expanded reference database of human and mouse transcriptional regulatory interactions. Nucleic Acids Res. 2018;46(D1):D380-D386. 10.1093/nar/gkx1013. 10.1093/nar/gkx1013 PMC5753191 29087512

[21] Szklarczyk D, Kirsch R, Koutrouli M (2023). The STRING database in 2023: protein-protein association networks and functional enrichment analyses for any sequenced genome of interest. Nucleic Acids Res.

[22] Cerami EG, Gross BE, Demir E, et al. Pathway Commons, a web resource for biological pathway data. Nucleic Acids Res. 2011;39(Database issue):D685–690. 10.1093/nar/gkq1039. 10.1093/nar/gkq1039 PMC3013659 21071392

[23] Hu H, Miao YR, Jia LH, et al. AnimalTFDB 3.0: a comprehensive resource for annotation and prediction of animal transcription factors. Nucleic Acids Res. 2019;47(D1):D33–D38. 10.1093/nar/gky822. 10.1093/nar/gky822 PMC6323978 30204897

[24] Rouillard AD, Gundersen GW, Fernandez NF, et al. The harmonizome: a collection of processed datasets gathered to serve and mine knowledge about genes and proteins. Database (Oxford).2016;2016:baw100. 10.1093/database/baw100. 10.1093/database/baw100 PMC4930834 27374120

[25] Li JH, Liu S, Zhou H, et al. starBase v2.0: decoding miRNA-ceRNA, miRNA-ncRNA and protein-RNA interaction networks from large-scale CLIP-Seq data. Nucleic Acids Res. 2014;42(Database issue):D92–7. 10.1093/nar/gkt1248. 10.1093/nar/gkt1248 PMC3964941 24297251

[26] Li R, Qu H, Wang S (2022). CancerMIRNome: an interactive analysis and visualization database for miRNome profiles of human cancer. Nucleic Acids Res.

[27] Dragotto J, Canterini S, Del Porto P (2019). The interplay between TGF-β-stimulated TSC22 domain family proteins regulates cell-cycle dynamics in medulloblastoma cells. J Cell Physiol.

[28] Nakamura M, Kitaura J, Enomoto Y (2012). Transforming growth factor-β-stimulated clone-22 is a negative-feedback regulator of Ras/Raf signaling: implications for tumorigenesis. Cancer Sci.

[29] Liang F, Li Q, Li X (2016). TSC22D2 interacts with PKM2 and inhibits cell growth in colorectal cancer. Int J Oncol.

[30] Ayroldi E, Cannarile L, Delfino DV (2018). A dual role for glucocorticoid-induced leucine zipper in glucocorticoid function: Tumor growth promotion or suppression?. Cell Death Dis.

[31] Hwang J, Haque MA, Suzuki H (2020). THG-1 suppresses SALL4 degradation to induce stemness genes and tumorsphere formation through antagonizing NRBP1 in squamous cell carcinoma cells. Biochem Biophys Res Commun.

[32] Roberts AW, Wei AH, Huang DCS (2021). BCL2 and MCL1 inhibitors for hematologic malignancies. Blood.

[33] Ong F, Kim K, Konopleva MY (2022). Venetoclax resistance: mechanistic insights and future strategies. Cancer Drug Resist.

[34] Xie X, Yang W, Zhang W, et al. Tegaserod maleate exhibits antileukemic activity by targeting TRPM8. Biomed Pharmacother. 2022;154:113566. 10.1016/j.biopha.2022.113566. 10.1016/j.biopha.2022.113566 35994820

[35] Redjimi N, Gaudin F, Touboul C (2009). Identification of glucocorticoid-induced leucine zipper as a key regulator of tumor cell proliferation in epithelial ovarian cancer. Mol Cancer.

[36] Ling Q, Li F, Zhang X, et al. MAP4K1 functions as a tumor promotor and drug mediator for AML via modulation of DNA damage/repair system and MAPK pathway. EBioMedicine. 2021;69:103441. 10.1016/j.ebiom.2021.103441. 10.1016/j.ebiom.2021.103441 PMC8239467 34166980

[37] Sun Y, Zhang D, Guo X (2019). MKK3 modulates JNK-dependent cell migration and invasion. Cell Death Dis.

[38] Wöss K, Simonović N, Strobl B (2019). TYK2: an upstream kinase of STATs in cancer. Cancers (Basel).

[39] Bi L, Jia S, Hu W (2022). Systematic analysis of prognostic significance, functional enrichment and immune implication of STK10 in acute myeloid leukemia. BMC Med Genom.

[40] Sugito N, Heishima K, Akao Y (2022). Chemically modified MIR143-3p exhibited anti-cancer effects by impairing the KRAS network in colorectal cancer cells. Mol Ther Nucleic Acids.

[41] Ronchetti S, Migliorati G, Riccardi C (2015). GILZ as a mediator of the anti-inflammatory effects of glucocorticoids. Front Endocrinol (Lausanne).

[42] Ma Y, Yang H, Kroemer G (2019). Endogenous and exogenous glucocorticoids abolish the efficacy of immune-dependent cancer therapies. Oncoimmunology.

[43] Yang H, Xia L, Chen J (2019). Stress-glucocorticoid-TSC22D3 axis compromises therapy-induced antitumor immunity. Nat Med.

[44] Ayroldi E, Migliorati G, Bruscoli S (2001). Modulation of T-cell activation by the glucocorticoid-induced leucine zipper factor via inhibition of nuclear factor kappaB. Blood.

[45] Berrebi D, Bruscoli S, Cohen N (2003). Synthesis of glucocorticoid-induced leucine zipper (GILZ) by macrophages: an anti-inflammatory and immunosuppressive mechanism shared by glucocorticoids and IL-10. Blood.

[46] Lebson L, Wang T, Jiang Q (2011). Induction of the glucocorticoid-induced leucine zipper gene limits the efficacy of dendritic cell vaccines. Cancer Gene Ther.

[47] Bereshchenko O, Coppo M, Bruscoli S (2014). GILZ promotes production of peripherally induced Treg cells and mediates the crosstalk between glucocorticoids and TGF-beta signaling. Cell Rep.

[48] Yan M, Hu J, Yuan H (2021). Dynamic regulatory networks of T cell trajectory dissect transcriptional control of T cell state transition. Mol Ther Nucleic Acids.

[49] Tabe Y, Konopleva M (2015). Role of microenvironment in resistance to therapy in AML. Curr Hematol Malig Rep.

