# Supplementary material for: Comprehensive bioinformatic analysis of the expression and prognostic significance of TSC22D domain family genes in adult acute myeloid leukemia
Source: BMC Med Genomics. 2023 May 27;16:117. doi: 10.1186/s12920-023-01550-7 (PMC10224341; doi:10.1186/s12920-023-01550-7)
Supplement: Supplementary file 3 — Additional file 3: Table S1. Analysis of the expression, correlation, and survival prognosis of predicted kinases regulated by TSC22D3 using the UCSCXenaShiny and Harmonizome database. [file 12920_2023_1550_MOESM3_ESM.docx]

Supplementary Table S1 Analysis of the expression, correlation, and survival prognosis of predicted kinases regulated by TSC22D3 using the UCSCXenaShiny and Harmonizome database.

| Gene symbol | 1. Score by   Harmonizome | The expression in 173 TCGA-LAML tissues and 70 GTEx -Normal tissues | P-value | Pearson correlation with TSC22D3 in 173 TCGA-LAML | P-value | Survival prognosis in 161 TCGA-LAML | P-value |
| --- | --- | --- | --- | --- | --- | --- | --- |
| TGFBR2 | 4.09742884 | High | 3.1E-34 | 0.13 | 0.10 | NS | 0.89 |
| MAP4K1 | 3.17059125 | High | 3.1E-34 | 0.26 | 5.9E-04 | adverse | 1.1E-02 |
| RIPK4 | 3.05255262 | Low | 2.3E-07 | 0.01 | 0.94 | NS | 0.54 |
| MAP2K2 | 2.71746811 | Low | 6.7E-13 | 0.27 | 3.2E-04 | adverse | 1.2E-02 |
| TIE1 | 2.70574641 | High | 2.9E-30 | -0.08 | 0.30 | NS | 0.89 |
| MAP2K3 | 2.4001097 | High | 3.1E-34 | 0.27 | 3.6E-04 | adverse | 6.7E-03 |
| TEC | 2.08124371 | High | 3.4E-27 | 0.06 | 0.47 | NS | 0.29 |
| TXK | 1.9665623 | High | 3.1E-34 | 0.03 | 0.72 | NS | 0.26 |
| TYK2 | 1.93330054 | High | 9.1E-23 | 0.48 | 2.0E-11 | adverse | 2.1E-02 |
| EPHB1 | 1.92940058 | High | 1.3E-27 | -0.05 | 0.52 | NS | 0.5 |
| FGFR4 | 1.83781417 | Low | 3.1E-34 | 0.21 | 5.8E-03 | NS | 0.3 |
| ITK | 1.75499145 | High | 2.7E-34 | 0.05 | 0.54 | NS | 0.93 |
| BTK | 1.71194261 | High | 1.0E-27 | 0.27 | 3.0E-04 | NS | 7.6E-02 |
| BCKDK | 1.70686298 | NS | 0.26 | 0.35 | 2.6E-06 | adverse | 5.8E-03 |
| ERN1 | 1.65429047 | High | 1.0E-12 | 0.21 | 5.2E-03 | NS | 0.71 |
| TESK2 | 1.63658201 | High | 4.1E-15 | 0.25 | 1.1E-03 | NS | 0.96 |
| GRK7 | 1.61193246 | High | 1.6E-20 | 0.02 | 0.77 | NS | 6.6E-02 |
| ZAP70 | 1.58559006 | High | 3.1E-34 | 0.04 | 0.61 | NS | 0.55 |
| KIT | 1.58195512 | High | 2.0E-28 | -0.16 | 3.3E-02 | favorable | 1.2E-02 |
| STK10 | 1.54163872 | High | 3.1E-34 | 0.33 | 7.5E-06 | adverse | 1.1E-02 |

Table footnotes: NS, no significance.
